# A decision tree model for predicting intravenous immunoglobulin resistance and coronary artery involvement in Kawasaki disease

**DOI:** 10.1186/s12887-022-03533-6

**Published:** 2022-08-05

**Authors:** Jinwoon Joung, Jun Suk Oh, Jung Min Yoon, Kyung Ok Ko, Gyeong Hee Yoo, Eun Jung Cheon

**Affiliations:** 1 Department of Pediatrics, Myunggok Medical Research Center, Konyang University College of Medicine, 158 Gwanjeodong-ro, Seo-gu, Daejeon, 35365 Korea; 2 Department of Pediatrics, Soonchunhyang University Cheonan Hospital, Sonnchunhyang 6-gil, Dongnam-gu, Cheonan, 31151 Korea

**Keywords:** Coronary artery dilatation, Decision tree model, IVIG resistance, Kawasaki disease

## Abstract

**Objectives:**

This study aims to develop a new algorithm for predicting intravenous immunoglobulin (IVIG) resistance and coronary artery involvement in Kawasaki disease (KD) through decision tree models.

**Methods:**

Medical records of children hospitalized for KD were analysed retrospectively. We compared the clinical characteristics, and the laboratory data in the groups with IVIG resistance and coronary artery dilatations (CADs) in KD patients. The decision tree models were developed to predict IVIG resistance and CADs.

**Results:**

A total 896 patients (511 males and 385 females; 1 month-12 years) were eligible. IVIG resistance was identified in 111 (12.3%) patients, and CADs were found in 156 (17.4%). Total bilirubin and nitrogen terminal- pro-brain natriuretic peptide (NT-proBNP) were significantly higher in IVIG resistant group than in IVIG responsive group (0.62 ± 0.8 mg/dL vs 1.38 ± 1.4 mg/dL and 1231 ± 2136 pg/mL vs 2425 ± 4459 mL, respectively, *P* < 0.01). Also, CADs were more developed in the resistant group (39/111; 14.9% vs. 117/785; 35.1%, *P* < 0.01). The decision tree for predicting IVIG resistance was classified based on total bilirubin (0.7 mg/mL, 1.46 mg/dL) and NT-proBNP (1561 pg/mL), consisting of two layers and four nodes, with 86.2% training accuracy and 90.5% evaluation accuracy. The Receiver Operating Characteristic (ROC) evaluated the predictive ability of the decision tree, and the area under the curve (AUC) (0.834; 95% confidence interval, 0.675–0.973; *P* < 0.05) showed relatively higher accuracy. The group with CADs had significantly higher total bilirubin and NT-proBNP levels than the control group (0.64 ± 0.82 mg/dL vs 1.04 ± 1.14 mg/dL and 1192 ± 2049 pg/mL vs 2268 ± 4136 pg/mL, respectively, *P* < 0.01). The decision trees for predicting CADs were classified into two nodes based on NT-proBNP (789 pg/mL) alone, with 83.5% training accuracy and 90.3% evaluation accuracy.

**Conclusion:**

A new algorithm decision tree model presents for predicting IVIG resistance and CADs in KD, confirming the usefulness of NT-proBNP as a predictor of KD.

## Introduction

Kawasaki disease (KD) is an acute febrile systemic vasculitis of children, which is effectively treated with intravenous immunoglobulin (IVIG) and aspirin [1, 2]. However, despite the appropriate treatment modulating inflammatory response, coronary artery dilatations (CADs) still occur in 15–20% of patients with KD [3, 4]. Furthermore, CADs, the potential lesion to develop stenosis or obstructions, are children's leading cause of acquired heart disease [4–8]. Non-responsiveness to IVIG treatment has been considered a sentinel of poor prognosis in patients with KD. Therefore, early risk stratification for IVIG resistance and intensifying initial therapy for those patients is essential for reducing adverse outcomes [9–15]. KD is a self-limited disease regardless of severity. Earlier studies in the pre-IVIG era reported that the mean fever duration of KD was 10–11 days [16]. Thus, it is proposed that the intensity of systemic inflammation gradually increases and reaches the peak (mean at the sixth febrile day), then gradually decreases and enters the convalescent stage during the febrile stage of KD. The intensity of systemic inflammation during this process is reflected in laboratory parameters including C-reactive protein (CRP), white blood cell count (WBC) with neutrophil and lymphocyte (N/L) differential, albumin, hemoglobin, platelet (PLT), immunoglobulins, and other cytokines and chemokines. The findings suggest that the immune reaction of the host before the peak of inflammation may be responsible for tissue cell injury. Thus, early control of inflammation is crucial for reducing tissue cell injury and morbidity of the disease. The IVIG-non-responders may have severe systemic inflammation, which is in part controlled by initial IVIG [17]. Although there was no consensus on the definition of IVIG-non-responsiveness or refractory KD, early treatment is a significant subject for severely affected patients with KD. The effect of immune modulators is dependent on the severity of systemic inflammation of KD and the immune status of the hosts [18].

Researchers have attempted to use the acute inflammatory parameters described above to predict IVIG non-responsiveness [10–15]. Several scoring systems which were consisted of such factors were suggested. Kobayashi score has been the most widely used, and Egami score and Sano score are also used. However, the results of these models are uneven and have failed to be applicable across the population [19, 20]. Also, they are considered quite cumbersome and underutilized in clinical practice due to the complex process as categorizing several factors, scoring points, and adding them to the total score.

Recently, a decision tree model creating an algorithm that resembles a human thought process has been considered a proper statistical method for developing clinical prediction models [21]. By decision tree analysis, we aimed to develop an easy-to-use prediction model for IVIG resistance and coronary artery involvement in patients with KD.

### Patients

We reviewed the medical records of all consecutive patients with KD who were hospitalized in two tertiary centers (Konyang University Hospital and Soonchunhyang University Hospital) from January 2015 to June 2019. Nine hundred and ninety-four children were hospitalized for KD during the period. Only those who fulfilled the diagnostic criteria for KD were included in this analysis, and patients with missing values were excluded. A total of 896 patients were included in this study.

## Methods

### Diagnosis and treatment of Kawasaki disease

The diagnosis and treatment of the two hospitals were conducted on the same principle based on American Heart Association guideline [22] which included fever accompanied by at least four of the following five findings: bilateral conjunctival injection, changes in the lips and oral cavity, non-purulent cervical lymphadenopathy, polymorphous exanthema, and changes in the extremities. Initial treatment was as follows; IVIG (2 g/kg/day) infusion was done over 10–12 h with 50 mg/kg of aspirin. Laboratory tests were performed at the time of admission and 48 h after IVIG treatment. A patient was considered afebrile when body temperature remained below 37.5 °C for more than 24 h.

### The clinical and laboratory findings

We collected the data at the time of admission. The clinical data include sex, age, days of illness, IVIG responsiveness, and CADs. The laboratory data include complete blood count (CBC), absolute neutrophil count (ANC), haematocrit, platelet, CRP, aspartate aminotransferase (AST), Alanine aminotransferase (ALT), total bilirubin, albumin, serum sodium level, PLT and nitrogen terminal- pro brain natriuretic peptide (NT-proBNP).

### IVIG resistance and coronary artery dilations

IVIG resistance is defined as persisting fever (body temperature above 38.0℃) for over 36 h after completion of IVIG (2 g/kg). Most of resistant patients were given the second dose of IVIG. A few IVIG resistant patients received methylprednisolone pulse therapy (15-30 mg/kg) for three days, depending on clinical decisions. CADs are defined as (1) internal luminal diameters over 3 mm for ≤ 5 years old, or 4 mm for > five years old, or (2) ≥  + 2.5 SD for a patient’s body surface area (BSA).

### Statistical analysis

Collected data were analysed using IBM SPSS Statistics ver. 26.0 (IBM Co., Armonk, NY, USA). Normality of distribution was tested by use of the Kolmogorov–Smirnov test. Comparison between categorical data was done by χ2 test, and continuous variables were compared using Student’s *t-*test. We accomplished recursive partitioning analysis to develop prediction models for IVIG resistance and the development of CADs. To avoid overfitting, all possible cross-tabulations for each categorical predictor have been created until the best outcome achieves no further splitting. The area under the receiver operating characteristics curve (ROC) analysis was conducted to evaluate the predictive competence of the decision tree predictive model. All significance tests used a *P* value < 0.05.

## Results

### Demographics and baseline characteristics

511 boys and 385 girls were included in the study. Patients’ age ranged from 1 month to 12 years (median, 25 months). The duration of fever before admission was 4 days (2–13 days). A total of 111 (12.3%) patients were resistant to initial IVIG treatment so they needed additional IVIG, and 156 (17.4%) patients developed CADs. 45 children received methylprednisolone pulse therapy (15-30 mg/kg for three consecutive days) to control fever.

### Decision tree model for predicting IVIG resistance

IVIG resistance was identified in 111 (12.3%) patients, and CADs were found in 156 (17.4%). IVIG non-responders had higher ANC, AST, ALT, CRP, total bilirubin and NT-proBNP values. Platelet was shown to be lower. Also, CADs were more prevalent in the resistant group (39/111; 14.9% vs. 117/785; 35.1%, *P* < 0.01) (Table 1).

**Table 1:** The Characteristics of patients with Kawasaki disease according to IVIG responsiveness

	**IVIG responders (*N* = 117)**	**IVIG non-responders (*N* = 785)**	***P* value**
Age, months	31.43 ± 22.44	36.39 ± 27.70	0.07
Sex			
Male, n (%)	444 (56.5%)	67 (60.3%)	0.47
Female, n (%)	341 (43.4%)	44 (39.6%)	
WBC, 10^3^/L	14.8 ± 4.6	15.2 ± 6.0	0.36
ANC, 10^3^/L	9.56 ± 4.4	11.36 ± 5.3	< 0.01*
Hematocrit, %	34.3 ± 12.7	35.2 ± 5.7	0.43
Platelet, 10^3^/L	361.46 ± 100.66	322.14 ± 105.44	< 0.01*
AST, U/L	79.8 ± 138.8	135.6 ± 201.5	< 0.01*
ALT, U/L	79.7 ± 127.9	159.1 ± 188.3	< 0.01*
Albumin, g/dL	3.97 ± 0.9	3.80 ± 0.5	0.06
Total bilirubin, mg/dL	0.62 ± 0.8	1.38 ± 1.4	< 0.01*
CRP, mg/dL	8.02 ± 5.9	9.92 ± 6.3	< 0.01*
Na, mmol/L	136.3 ± 2.8	135.8 ± 2.5	0.09
NT-proBNP, pg/mL	1231.9 ± 2136.5	2425.4 ± 4459.3	< 0.01*
Development of CADs, n (%)	39 (14.9%)	117 (35.1%)	< 0.01*

mean ± standard deviation or number (%). *means statistically significant; *p* values < 0.05

*IVIG* Intravenous immunoglobulin, *WBC* White blood cells, *ANC* Absolute neutrophil counts, *AST* Aspartate aminotransferase, *ALT* Alanine transaminase, *CRP* C-reactive protein, *NT-proBNP* Nitrogen terminal-pro brain natriuretic peptide, *CAD* Coronary artery dilatation

**Fig. 1:**
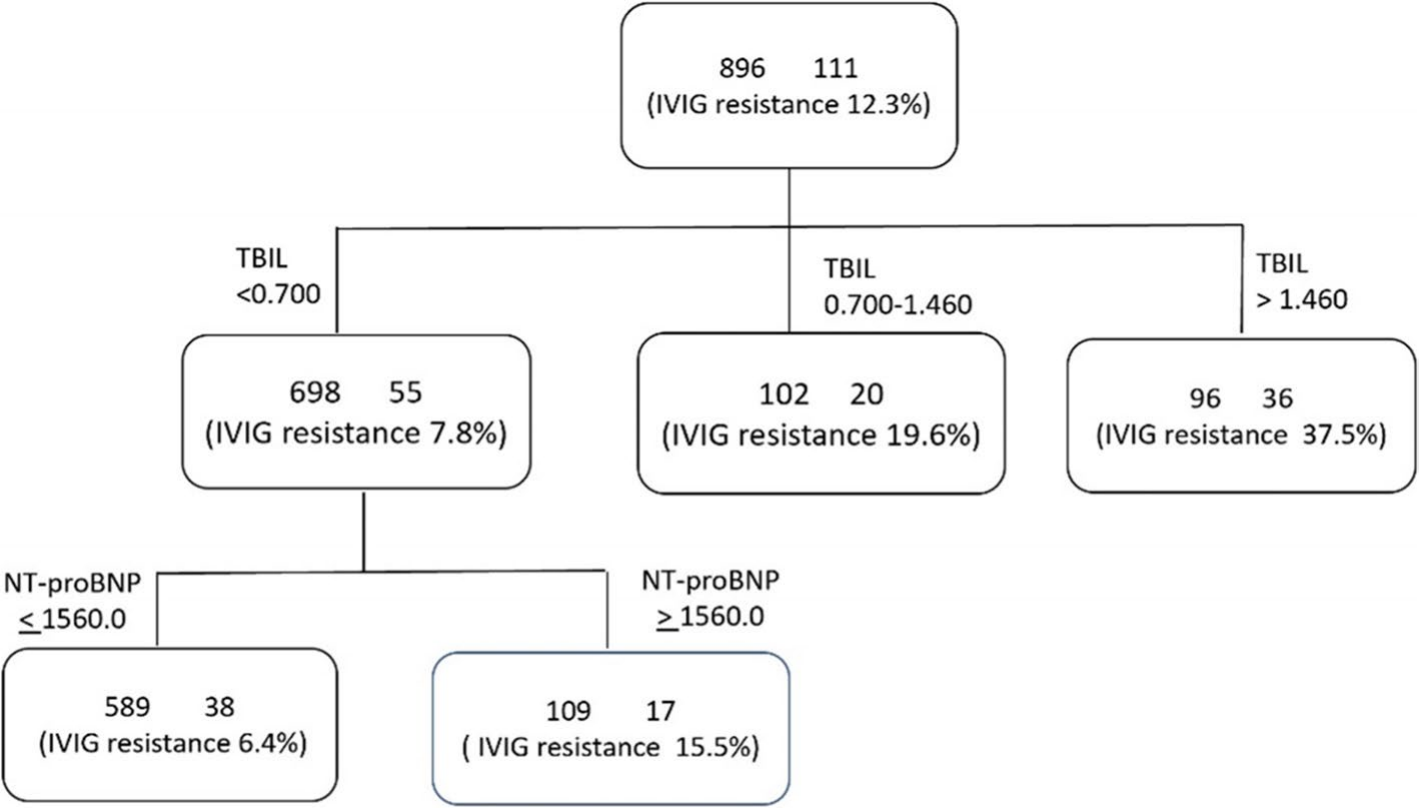
Decision Tree Model for predicting IVIG resistance in Kawasaki disease. IVIG, intravenous immunoglobulin, TBIL, total bilirubin; NT-proBNP, nitrogen terminal-brain natriuretic peptide

A decision tree model for predicting IVIG non-responsiveness was generated into two layers and four nodes. Total bilirubin was the most important discriminating factor, followed by NT-proBNP (Fig. 1).

Patients with total bilirubin levels higher than 1.46 mg/dL had the highest risk of IVIG resistance. Others with total bilirubin levels lower than 0.7 mg/dL and NT-proBNP lower than 1561.0 pg/mL concurrently had the lowest risk. The decision tree model for IVIG resistance had a training accuracy of 86.2% and a test accuracy of 90.5%.

**Fig. 2:**
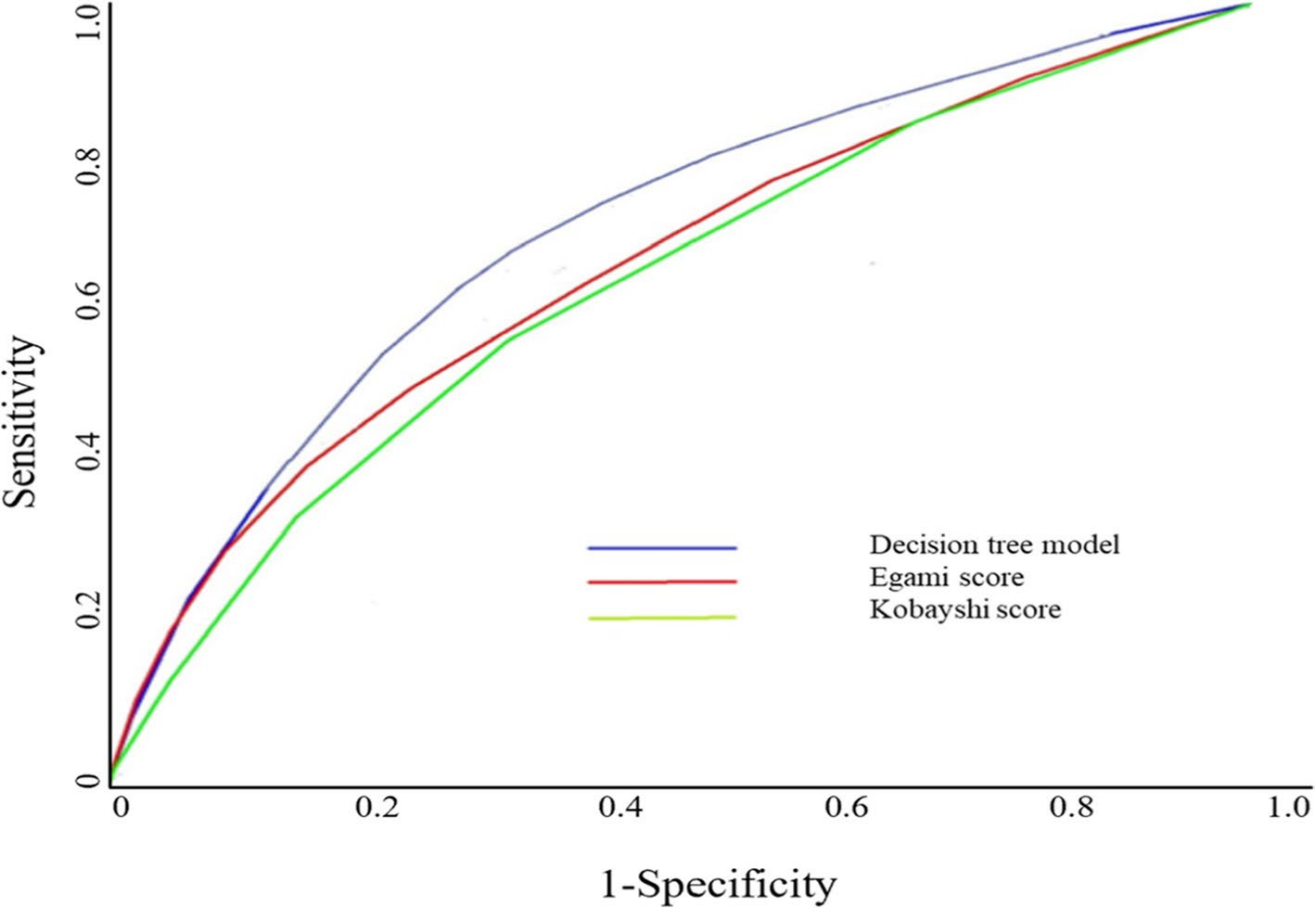
ROC curves of decision tree model for IVIG resistance in patients with Kawasaki disease. ROC, Receiver Operating Characteristic; IVIG, intravenous immunoglobulin

ROC-AUC was used to evaluate the predictive abilities of the decision tree models. Previously, several risk scoring systems of IVIG resistance in KD have been published [12, 13, 19]. Of all the pre-existing scores, Egami score (ES) [13] and Kobayashi score (KS) [12] were compared with current decision tree models (Fig. 2). The AUC was 0.834 (95% CI [0.675–0.973]), which is relatively accurate than ES and KS.

### Decision tree model for predicting CADs

The serum level of total bilirubin (1.04 ± 1.14, *P* < 0.01) and NT-proBNP (2268.5 ± 4136.7, *P* < 0.01) were significantly higher in CADs group. Patients with CAD also had higher ANC, ALT, and CRP values. Albumin and serum sodium was shown to be lower in the group with CADs (Table 2).

**Table 2:** The characteristics of patients with Kawasaki disease according to development of CADs

	**CADs (-) *N* = 740**	**CADs ( +) *N* = 156**	***P* value**
Age, months	31.86 ± 22.54	32.92 ± 26.15	0.60
Sex			0.53
Male, n (%)	418 (56.4%)	93 (59.6%)	
Female, n (%)	322 (43.5%)	63 (40.3%)	
WBC, 10^3^/L	14.73 ± 4.75	15.22 ± 5.10	0.25
ANC, 10^3^/L	9.59 ± 4.44	10.71 ± 4.87	< 0.01
Hematocrit, %	34.40 ± 13.06	34.33 ± 5.08	0.94
Platelet, 10^3^/L	358.78 ± 101.58	346.15 ± 103.82	0.16
AST, U/L	83.23 ± 144.13	103.38 ± 169.70	0.12
ALT, U/L	81.05 ± 130.57	129.67 ± 169.03	< 0.01*
Albumin, g/dL	3.97 ± 0.95	3.82 ± 0.44	0.04
Total bilirubin, mg/dL	0.64 ± 0.82	1.04 ± 1.14	< 0.01*
CRP, mg/dL	7.94 ± 5.68	9.77 ± 6.85	< 0.01*
Na, mmol/L	136.37 ± 2.71	135.67 ± 3.01	< 0.01*
NT-proBNP, pg/mL	1192.48 ± 2049.08	2268.57 ± 4136.75	< 0.01*
IVIG resistance, n (%)	72 (9.7%)	39 (25.0%)	< 0.01*

mean ± standard deviation or number (%). *means statistically significant; *p* values < 0.05

*IVIG* Intravenous immunoglobulin, *WBC* White blood cells, *ANC* Absolute neutrophil counts, *AST* Aspartate aminotransferase, *ALT* Alanine transaminase, *CRP* C-reactive protein, *NT-proBNP* Nitrogen terminal- pro brain natriuretic peptide, *CAD* Coronary artery dilatation

**Fig. 3:**
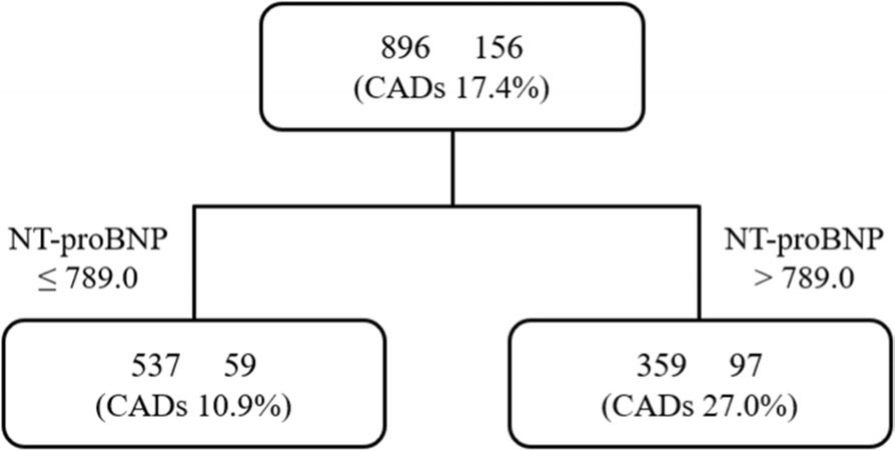
Decision tree model for predicting coronary artery dilations in Kawasaki disease. CADs; coronary artery dilatation; NT-proBNP, nitrogen terminal-brain natriuretic peptide

Based on recursive partitioning analysis, a decision tree model for predicting CADs in KD was built into one layer and two nodes. The analysis identified NT-proBNP as the most valuable predictor (Fig. 3). Patients with NT-proBNP higher than 789.0 pg/mL had a higher risk of developing CADs. The decision tree model for CADs shows a training accuracy of 83.5% test accuracy of 90.3%.

## Discussion

We developed an easy-to-use prediction model for IVIG resistance and coronary artery involvement in patients with KD. Our predictive model can distinguish high-risk patients without going through the process of categorizing or scoring the various factors. As far as we know, this study is the first to suggest an algorithm based on a decision tree model predicting the risk of IVIG resistance and the development of CADs.

A decision tree model simplifies complex relationships between parameters by dividing original input variables into statistically significant subgroups. The decision tree method does not need data transformation to handle skewed data and there is no need for imputation to handle missing values. It is very similar to the process of human thought and is easy to understand and interpret. The method is an intuitive and non-parametric approach without distributional assumptions.

KD and consequent coronary artery lesions are the leading causes of acquired heart disease in children and are further relevant to late sequelae in the young adult phase [4–8]. Therefore, offering intensified initial treatment for patients with a high risk of developing CADs or IVIG resistance is clinically essential. However, predicting IVIG responsiveness or the cardiac prognosis in the early disease course remains challenging. Several predicting factors and risk scoring systems for IVIG resistance and CADs development have been presented [10–15]. The Kobayashi score, the most widely used scoring system, has been used as a standard indicator for determining high-risk patients in random prospective studies using cyclosporin [23] and steroids [24]as initial intensification treatments. It is a seven-variable model constructed, day of illness at initial treatment, age in months, percentage of white blood cells representing neutrophils, platelet count, serum aspartate aminotransferase, sodium, and C-reactive protein. However, the levels of WBC count with N/L differs by fever duration at presentation in the early stage of KD. Also, some parameters such as haemoglobin and albumin have variable normal values according to ages in childhood. They are also influenced by the different composition of patients' age, fever duration at presentation, and organ involvement in every studies. Accordingly, the scoring systems for severe cases, including CRP and WBC, might fail to be useful across populations even in the same nations, Japan and China [25].

The decision tree model with a single predictor, NT-proBNP, can easily estimate the risk of developing CADs. The other decision tree model, composed of total bilirubin and NT- proBNP, can accurately predict the risk of IVIG resistance.

Myocardial involvement in an acute febrile phase of KD has been widely recognized [17]. Several studies have represented the predictive value of NT-proBNP for differentiating KD from other febrile illnesses and predicting the prognosis of the disease. According to those previous studies, a higher level of NT-proBNP indicates a higher risk of IVIG resistance and CADs, but still, there is no standardized cut-off value [25, 26]. Endomyocardial biopsies of most KD patients show inflammatory infiltrates, necrosis, and fibrosis of myocytes, along with immunoreactivity for BNP [27]. An active form of proBNP cleavage, BNP is released when the ventricular wall is stressed with volume or pressure overload. Hence elevated BNP level allows detection of myocardial involvement in the early stage of KD. Unlike BNP, NT-proBNP, an inactive form of proBNP cleavage, is elevated even before hemodynamic instability with a longer half-life. Also, NT-proBNP is less affected by patients' renal functions and chemically more stable at room temperature so that the serum level might be more precise. These traits make NT-proBNP much more helpful in clinical use compared with BNP. Prior studies have recognized NT-proBNP as a valuable marker for diagnosing KD [25, 26]. However, only a few studies have explored the value of NT-proBNP as a part of a diagnostic algorithm or risk scoring system in KD. The clinical application of NT-proBNP was limited to adjunctive tests, insufficient for a stand-alone test [25, 28, 29]. Meanwhile, Dionne et al. [28] presented an NT-proBNP-based algorithm for identifying incomplete KD and proved it superior to the 2004 American Heart Association algorithm. Likewise, NT-proBNP levels principally have predicted the possibility of both IVIG resistance and the development of CADs in the decision tree models.

Additionally, total bilirubin implicating liver dysfunction in KD was our study's most critical factor for IVIG resistance [30]. As is well known, the liver is commonly involved in KD. It appears to be an elevation of AST, ALT, and an increase in total bilirubin means more severe liver involvement. Recently, Fang et al. reported that KD patients with hyperbilirubinemia did not respond well to initial IVIG treatment and had more CADs at the time of hospitalization. The physiopathology of hyperbilirubinemia and cholestatic liver damage in KD remains undetermined. Vasculitis-associated inflammation and obstruction in the liver and gallbladder might be the cause of increased transaminase levels and cholestasis [31].

We supposed that in KD, various cytokines cause systemic inflammatory reactions, of which interleukin-6 stimulates liver cells to produce CRP, activating the complements and increasing phagocytosis of white blood cells. Therefore, the more severe the systemic inflammatory response, the higher the likelihood of developing hyperbilirubinemia [32].

However, there are a few limitations in our study. KD is a heterogeneous syndrome presenting wide range of severity from mild to severe cases. Moreover, organ involvements such as CADs and liver involvement are observed in some KD patients. Thus, some indices such as Na, bilirubin, AST, and ALT cannot be generalized for all KD. Also, since this is a two-center study, patient demographic data may differ from other institutions. Finally, age-adjusted serum NT-proBNP levels might have made more delicate outcomes [33]. Large-scale prospective studies would further validate and sharpen the clinical applicability of the decision tree models.

## Conclusion

Based on a decision tree model study, a new algorithm precisely predicts the risk of IVIG resistance and the development of CADs in KD. We propose NT-proBNP as an excellent prognostic marker in patients with KD.
